# Metal artifact reduction in ultra-high-resolution cone-beam CT imaging with a twin robotic X-ray system

**DOI:** 10.1038/s41598-022-19978-9

**Published:** 2022-09-16

**Authors:** Andreas Steven Kunz, Theresa Sophie Patzer, Jan-Peter Grunz, Karsten Sebastian Luetkens, Viktor Hartung, Robin Hendel, Tabea Fieber, Franca Genest, Süleyman Ergün, Thorsten Alexander Bley, Henner Huflage

**Affiliations:** 1 Department of Diagnostic and Interventional Radiology, University Hospital Würzburg, Oberdürrbacher Straße 6, 97080 Würzburg, Germany; 2 Department of Trauma, Hand, Plastic and Reconstructive Surgery, University Hospital Würzburg, Oberdürrbacher Straße 6, 97080 Würzburg, Germany; 3 Orthopedic Clinic König-Ludwig-Haus, Julius-Maximilians-Universität Würzburg, Brettreichstr. 11, 97070 Würzburg, Germany; 4 Institute of Anatomy and Cell Biology, University of Würzburg, Koellikerstr. 6, 97070 Würzburg, Germany

**Keywords:** Medical research, Preclinical research

## Abstract

Cone-beam computed tomography (CBCT) has been shown to be a powerful tool for 3D imaging of the appendicular skeleton, allowing for detailed visualization of bone microarchitecture. This study was designed to compare artifacts in the presence of osteosynthetic implants between CBCT and multidetector computed tomography (MDCT) in cadaveric wrist scans. A total of 32 scan protocols with varying tube potential and current were employed: both conventional CBCT and MDCT studies were included with tube voltage ranging from 60 to 140 kVp as well as additional MDCT protocols with dedicated spectral shaping via tin prefiltration. Irrespective of scanner type, all examinations were conducted in ultra-high-resolution (UHR) scan mode. For reconstruction of UHR-CBCT scans an additional iterative metal artifact reduction algorithm was employed, an image correction tool which cannot be used in combination with UHR-MDCT. To compare applied radiation doses between both scanners, the volume computed tomography dose index for a 16 cm phantom (CTDI_vol_) was evaluated. Images were assessed regarding subjective and objective image quality. Without automatic tube current modulation or tube potential control, radiation doses ranged between 1.3 mGy (with 70 kVp and 50.0 effective mAs) and 75.2 mGy (with 140 kVp and 383.0 effective mAs) in UHR-MDCT. Using the pulsed image acquisition method of the CBCT scanner, CTDI_vol_ ranged between 2.3 mGy (with 60 kVp and 0.6 mean mAs per pulse) and 61.0 mGy (with 133 kVp and 2.5 mean mAs per pulse). In essence, all UHR-CBCT protocols employing a tube potential of 80 kVp or more were found to provide superior overall image quality and artifact reduction compared to UHR-MDCT (all *p* < .050). Interrater reliability of seven radiologists regarding image quality was substantial for tissue assessment and moderate for artifact assessment with Fleiss kappa of 0.652 (95% confidence interval 0.618–0.686; *p* < 0.001) and 0.570 (95% confidence interval 0.535–0.606; *p* < 0.001), respectively. Our results demonstrate that the UHR-CBCT scan mode of a twin robotic X-ray system facilitates excellent visualization of the appendicular skeleton in the presence of metal implants. Achievable image quality and artifact reduction are superior to dose-comparable UHR-MDCT and even MDCT protocols employing spectral shaping with tin prefiltration do not achieve the same level of artifact reduction in adjacent soft tissue.

## Introduction

In postoperative follow-up after joint arthroplasty, plain radiography is the primary imaging method due to ubiquitous availability, cost-efficiency and fast imaging results while pertaining relatively low radiation doses. For a more detailed analysis of suspected complications after surgery, additional CT may be necessary, albeit being associated with a higher dose penalty. However, in postoperative settings, artifacts caused by metal implants may hamper diagnostic accuracy for assessment of the implant itself, the implant-bone interface, as well as its adjacent tissue^1,2^. Typical metal artifacts include beam hardening and photon starvation: beam hardening occurs when polychromatic X-ray photons pass through dense objects resulting in stronger absorption of low energy photons, which causes hyperdense artifacts with adjacent dark streaks. In contrast, photon starvation artifacts manifest due to complete absorption of photons, which leads to hypodense streaks^3–5^. As a result, detecting complications in the presence of metal implants, such as secondary dislocation, areas of bone resorption or implant loosening as indicated by a surrounding radiolucent rim or even fluid collections in the soft tissue, can pose a significant challenge.

Different approaches for metal artifact reduction (MAR) have been evaluated predominantly for conventional gantry-based, multidetector CT (MDCT) scanners in the past^6,7^. Photon starvation can be diminished by increasing the tube current in order to augment the number of photons in the X-ray beam. Increased tube voltage and thus higher photon energy leads to a higher penetration rate of dense material. At higher tube voltages, image noise and photon starvation can be minimized at the cost of decreased tissue contrast. However, reduction of metal artifacts at the expense of higher radiation doses is debatable, especially in young patients and patients with recurring examinations^8^. A similar effect can be seen when employing tin prefiltration, which increases photon penetration by reducing the amount of low-energy photons and therefore hardening the X-ray-beam^7,9^. While these protocol-based MAR approaches need to be established in advance of image acquisition, algorithms such as iterative reconstruction techniques can be executed retrospectively without negatively effecting the radiation dose. On the downside, iterative reconstruction algorithms may introduce secondary artifacts and have been reported to alter image information in general^10,11^. Furthermore, image data may be lost near the metal edge by interpolation^12^. Apart from optimization of metal implants and scan protocols, other approaches to reduce suchlike artifacts include model-based data correction, and image-based postprocessing^13^.

Due to the excellent image quality of bone tissue at relatively low dose, cone-beam CT (CBCT) has more recently developed a growing niche in musculoskeletal imaging. In trauma assessment, CBCT has already emerged as a viable alternative for depiction of the upper and lower extremity^14–18^. In a post-treatment setting, however, the value of non-dental CBCT systems has not been thoroughly investigated thus far. Especially in patients with limited mobility after osteosynthesis, the prone position and arm elevation required for optimized imaging conditions in wrist scans can be problematic with gantry-based MDCT scanners. Addressing this limitation with the investigated gantry-free multi-purpose scanner's option for tableside positioning of the upper extremity can be advantageous with regards to image quality and radiation dose^19^.

The goal of this study was to investigate the metal artifact reduction capabilities of a twin robotic X-ray system with an ultra-high-resolution (UHR)-CBCT scan mode in an experimental setting. Therefore, we compared the scanner’s performance to UHR-MDCT in cadaveric wrist scans after arthroplasty, aiming to find the best compromise between radiation dose and image quality.

## Material and methods

Our study was approved by the Institutional Review Board of the University of Würzburg, Germany. During their lifetime, donors volunteered their body to the university’s anatomical institute for study and research purposes. Additional written informed consent was not required.

All methods were performed in accordance with the relevant guidelines and regulations. The datasets used and/or analysed during the current study are available from the corresponding author on reasonable request.

### Cadaveric specimens and wrist positioning

Two trauma surgeons performed volar locking plate fixation (Aptus, Medartis, Basel, Switzerland) on a formalin-fixed cadaveric specimen’s distal radius. The wrist was then examined employing a multipurpose, twin robotic X-ray system for CBCT imaging and a high-end MDCT scanner. MDCT studies were performed in the so-called “superman stance” with the specimen in prone position and elevation of the respective arm above the head. CBCT studies were conducted in supine position with the upper extremity abducted at a 90° angle for a tableside scan position.

### Scanners

The employed multi-use X-ray system with implemented 3D CBCT mode (Multitom Rax, Siemens Healthineers, Erlangen, Germany) is equipped with two telescopic arms carrying the X-ray tube and flat-panel detector and is connected to ceiling rails with three translational and two rotational degrees of freedom. The X-ray tube is capable of providing currents from 0.5 to 800 mAs and voltages from 40 to 150 kV. The input field of the flat panel detector measures 23 cm × 23 cm with a 3D matrix of 1440 × 1440 un-binned pixels, which results in an effective pixel size of 148 μm. At the same time, the input field limits the CBCT’s maximum field of view since table movement and helical acquisition are not feasible with this system. For the acquisition of 3D projection data in CBCT scan mode, the arms move simultaneously and in synchronized fashion along predefined scanning trajectories. The tableside trajectory for upper extremity imaging has a sweep angle of 200° around the isocentre with an asymmetric source-to-image distance of 115 cm (tube-to-isocentre distance 85 cm, detector-to-isocentre distance 30 cm). For the purpose of the study, the wrist was additionally scanned with a high-end gantry-based MDCT scanner and multiple protocols in single-energy mode (Somatom Force, Siemens Healthineers).

### Scan protocols

Different scan protocols were evaluated while varying prefiltration, tube voltage and tube current-exposure time products. Thirty-two scans of the cadaveric wrist with volar locking plate fixation were performed in UHR mode. A total of 15 UHR-CBCT scans were evaluated with tube voltage ranging from 60 to 133 kVp and tube current-exposure-time products of 0.6 mAs, 1.2 mAs and 2.5 mAs. Of note, the CBCT system’s standard hardware does not include the option for tin prefiltration. The protocols for matching UHR-MDCT scans (15 matching MDCT protocols) were set up with tube voltage ranging from 70 to 140 kVp with reference mAs designed to equal CTDI values of their CBCT counterparts. Additionally, two protocols (100 kVp and 150 kVp) with 0.4 mm tin prefiltration were employed, resulting in a total of 19 MDCT protocols. Detailed scan parameters for CBCT and MDCT protocols are summarized in Table 1. For comparison of applied radiation doses between CBCT and MDCT, the volume computed tomography dose index for a 16 cm phantom (CTDI_vol_) was evaluated. Dose-length products and CTDI_vol_ were noted to estimate the radiation dose in MDCT. CTDI_vol_-equivalent values for CBCT were computed by multiplying the dose-area product by a linear scaling factor provided by the manufacturer based on previous phantom measurements. The scaling factor was determined in advance for every combination of acquisition geometry, tube voltage and beam filtration (e.g. copper filter). The dose-length product was measured in five chambers of a conventional polymethyl methacrylate dosimetry phantom (IEC 60601-2-44:2009) with a diameter of 160 mm, a length of 300 mm and a standard dosimetry system (Nomex Dosimeter, PTW, Freiburg im Breisgau, Germany) with a 300 mm ionisation chamber. Standard weighting scheme for dose measurements was applied to acquire volume dose-length product values (DLP_vol_). CTDI_vol_ values were calculated by dividing DLP_vol_ values by the field of view in the z-direction, which is equivalent to the beam width. Finally, the scaling factor was computed by dividing CTDI_vol_ by dose-area product values. Dose-area products for all examinations were extracted from the automatically created scan report.

**Table 1:** Dosimetric comparison of scan protocols.

Scanner	Voltage [kVp]	Current–time product [mAs]	CTDI_vol_ [mGy]
**MDCT** Siemens SOMATOM Force		Effective per scan	
	70	50.0	1.3
	70	97.0	2.7
	80	122.0	5.1
	70	199.0	5.3
	Sn 100	898.0	7.5
	Sn 150	146.0	8.2
	100	113.0	9.8
	80	241.0	10.2
	120	101.0	14.1
	140	96.0	18.9
	100	227.0	19.5
	80	489.0	20.3
	120	202.0	28.1
	140	192.0	37.7
	100	453.0	39.3
	120	405.0	54.0
	140	383.0	75.2
**CBCT** Siemens MULTITOM Rax		Mean per pulse	
	60	0.6	2.3
	60	1.2	4.5
	80	0.6	4.5
	102	0.6	9.1
	60	2.5	9.5
	80	1.2	9.9
	117	0.6	13.6
	102	1.2	16.3
	80	2.5	17.4
	133	0.6	21.6
	117	1.2	23.6
	102	2.5	34.6
	133	1.2	36.3
	117	2.5	44.1
	133	2.5	61.0

CTDI_vol_, computed tomography dose index calculated for a 16 cm phantom; CBCT, cone-beam computed tomography; MDCT, multidetector computed tomography; Sn, 0.4 mm tin prefiltration.

### Image reconstruction parameters

Scanner-side raw data reconstruction was performed employing a dedicated high-resolution kernel for very sharp depiction of osseous structures (Ur77; Siemens Healthineers) following the clinical standard for post-processing of MDCT examinations at our institution. Multiplanar reconstructions were carried out for CBCT and MDCT using dedicated 3D processing software (syngo.via View&GO and syngo.via, both Siemens Healthineers). For reconstruction of UHR-CBCT scans additional iterative metal artifact reduction (MAR) was employed. Reconstruction parameters for axial, coronal and sagittal planes were set with slice thickness of 1.0 mm, increment of 0.5 mm, field of view of 80 mm, and image matrix of 1024 × 1024 pixels irrespective of dose protocol and scanner. Window width and level of 3000 and 1000 Hounsfield units (HU) were predefined for optimal bone depiction. However, readers were allowed to alter window settings as personally required.

### Subjective image analysis

Images were evaluated independently in randomized and blinded fashion using clinical picture archiving and communication software (Merlin, Phoenix-PACS, Freiburg im Breisgau, Germany) by seven radiologists with varying levels of experience in musculoskeletal imaging (ASK 8, TSP 2, KSL 7, VH 6, RH 4, HH 6 and JPG 5 years of experience). No time restriction was imposed for reading. The readers were asked to rate the image quality with regards to osseous and soft tissue as well as the extent of artifacts using a seven-point scale (7 = excellent/no artifacts, 6 = very good/near absence of artifacts, 5 = good/mild artifacts, 4 = satisfactory/moderate artifacts, 3 = fair, /considerable artifacts 2 = poor/severe artifacts, 1 = very poor/not diagnostic due to artifacts). Based on these results we calculated figure-of-merit (FOM) values for bone and artifact ratings in order to characterize scan protocol performance and to rank image quality relative to radiation dose using the following formula:

$$FOM = \left( {sum\,of\,ratings} \right)^{2} /CTDI_{vol.}$$

### Objective image analysis

Objective image analysis was performed by a radiologist with 5 years of experience in musculoskeletal imaging (JPG). Circular regions-of-interest (ROI) were manually positioned in surrounding air and soft tissue as reference measuring signal attenuation in mean HU. Artifacts were quantified by placing a ROI in the most pronounced hypo- and hyperdense artifact areas as well as in the artifact-impaired soft tissue. Artifact measurements were performed three times, respectively, and averaged to guarantee high measurement accuracy and data consistency.

### Statistical analyses

Dedicated software was used for descriptive statistics and data analyses (SPSS Statistics Version 28, IBM, Armonk, New York, USA). P values less than 0.05 were considered to indicate statistical significance. Kolmogorov–Smirnov tests were applied to analyze continuous data for normal distribution. If normally distributed, continuous data is presented as mean ± standard deviation. Means of normally-distributed variables were compared with one-way ANOVA and Dunnett-T3 post-hoc tests with *p* values adjusted for multiple comparisons. Mean ranks of categorical items were compared with Friedman tests and post-hoc analysis of homogenous subsets. Fleiss kappa was calculated to investigate the interrater reliability in subjective image quality assessment. Agreement was interpreted according to Landis and Koch (1.00–0.81 = almost perfect; 0.80–0.61 = substantial; 0.60–0.41 = moderate; 0.40–0.21 = fair; 0.20–0.00 slight; < 0.00 poor agreement)^20^.

## Results

### Comparing the radiation dose

Thirty-two scan protocols with varying prefiltration, tube voltage and current–time product were applied in this study (17 UHR-MDCT, 15 UHR-CBCT). Without automatic tube current modulation or tube potential control, the highest and lowest radiation doses achieved in conventional MDCT were 75.2 mGy (with 140 kVp and 383.0 effective mAs) and 1.3 mGy (with 70 kVp and 50.0 effective mAs). Using the pulsed image acquisition method of the CBCT scanner, CTDI_vol_ of ultra-low-dose scans was 2.3 mGy (with 60 kVp and 0.6 mean mAs per pulse), while the highest possible dose was 61.0 mGy (with 133 kVp and 2.5 mean mAs per pulse). CTDI_vol_ values are summarized in Table 1.

### Quantifying the artifact reduction

**Figure 1:**
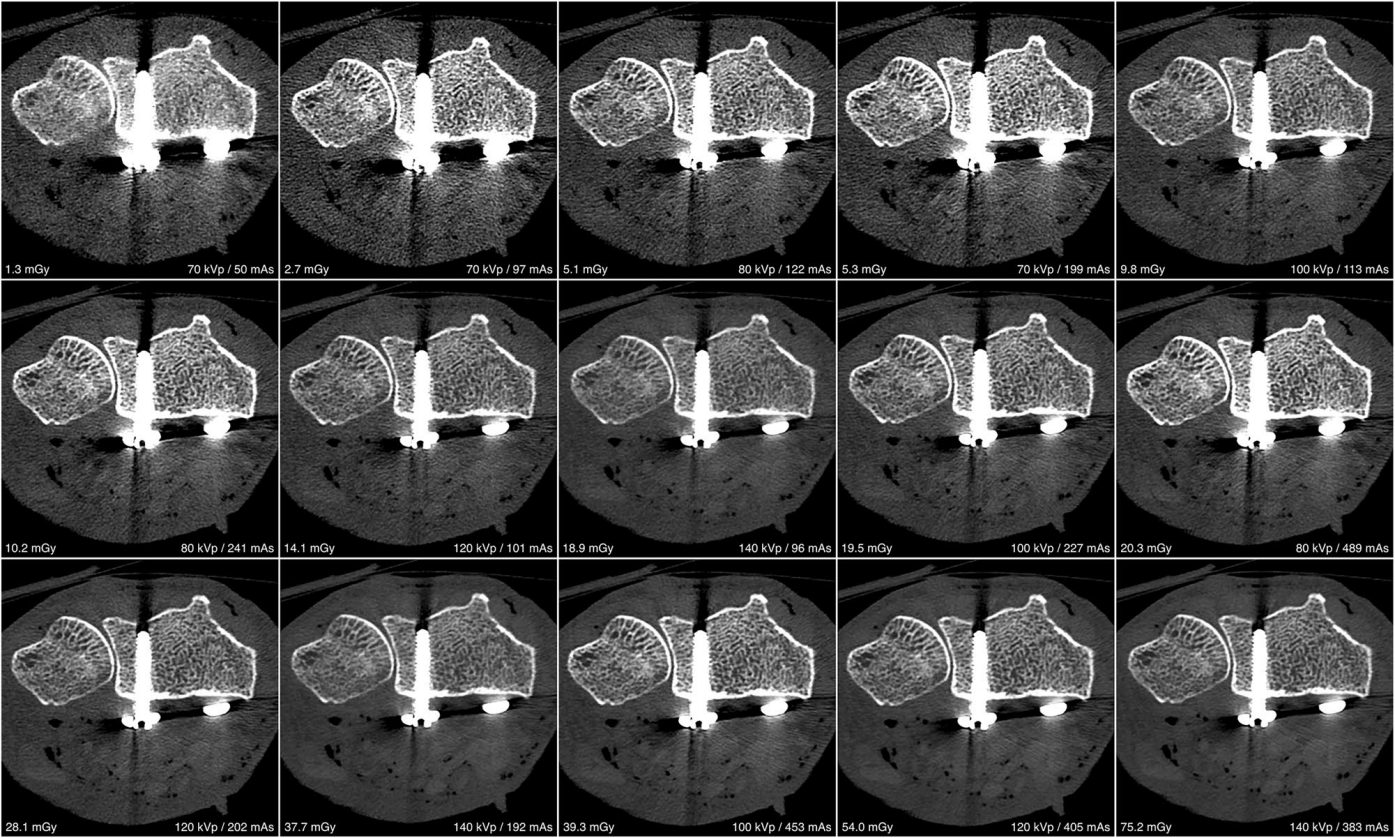
Bone image quality and intensity of metal artifacts by palmar plate osteosynthesis in conventional ultra-high-resolution MDCT scan protocols.

**Figure 2:**
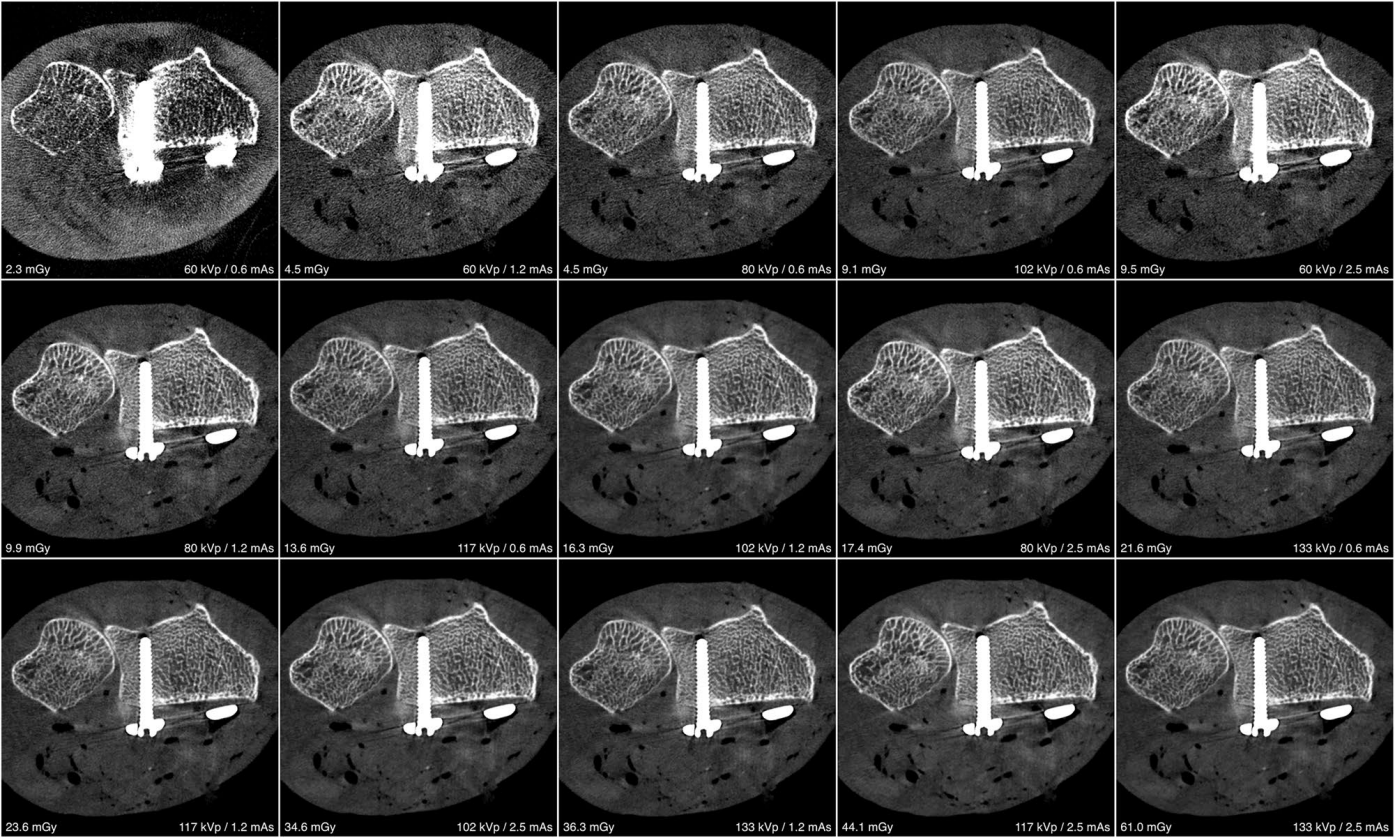
Bone image quality and intensity of metal artifacts by palmar plate osteosynthesis in ultra-high-resolution CBCT scan protocols.

**Figure 3:**
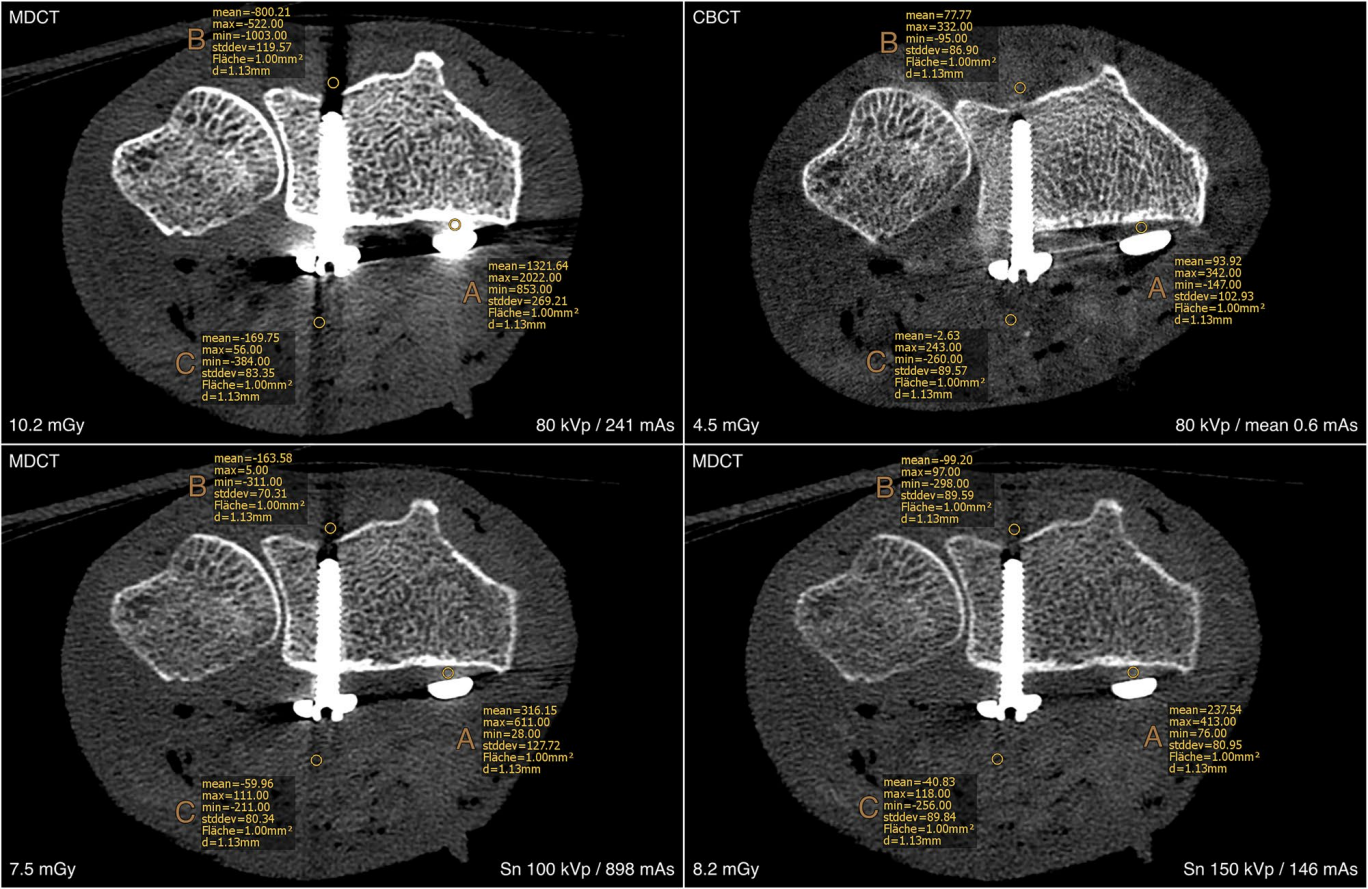
Region of interest (ROI) placement for objective assessment of metal artifact intensity. Note—A = hyperdense artifacts; B = hypodense artifacts; C = artifact-impaired soft tissue.

**Figure 4:**
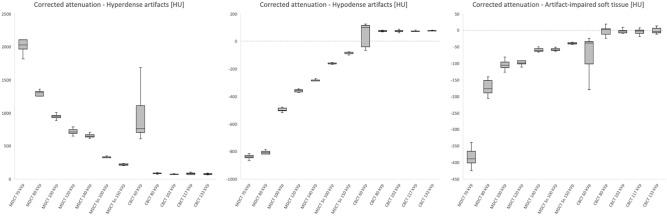
Boxplots with signal attenuation in artifacts and artifact-impaired soft tissue. Note—Boxplots (median and 50% of cases within the boxes) illustrate the corrected signal attenuation in Hounsfield units (HU) for hyperdense artifacts, hypodense artifacts and artifact-impaired soft tissue in cone-beam computed tomography (CBCT) and multidetector computed tomography (MDCT) with different levels of tube voltage (kVp = kilovoltage peak).

For visual comparison of image quality and metal artifact intensity with various combinations of tube potential and current on both scanners, representative images of the distal forearm after palmar plate osteoplasty are presented in Fig. 1 (UHR-MDCT) and Fig. 2 (UHR-CBCT). ROI-based quantification of signal attenuation in hyperdense beam hardening (all *p* < 0.036) and hypodense photon starvation artifacts (all *p* < 0.008) yielded favorable results for UHR-CBCT protocols with at least 80 kVp compared to UHR-MDCT imaging (Fig. 3). While providing inferior results in comparison with UHR-CBCT protocols, tin prefiltration lowered the extent of hyperdense and hypodense artifacts considerably compared to standard UHR-MDCT (all *p* < 0.003). With tin prefiltration activated, 150 kVp scans displayed stronger artifact reduction than 100 kVp imaging (*p* = 0.034). Impairment of adjacent soft tissue by artifacts was also reduced significantly with tin-prefiltered scan protocols (all *p* < 0.029), albeit UHR-CBCT with 80 kVp or more allowed for the best depiction of artifact-impaired soft tissue in this study (all *p* < 0.001). No significant differences were ascertained between UHR-CBCT scan protocols with at least 80 kVp (all *p* > 0.366). Results of objective artifact intensity assessment for both scanners are summarized in Table 2. In addition, boxplot diagrams are provided to illustrate the differences between UHR-MDCT and UHR-CBCT for each type of artifact (Fig. 4).

**Table 2:** Semiquantitative assessment of artifact reduction.

Corrected attenuation ± standard deviation	Hyperdense artifacts	Hypodense artifacts	Artifact impaired soft tissue
**MDCT**			
70 kVp	2070.3 ± 595.0	− 839.9 ± 140.0	− 385.4 ± 198.4
80 kVp	1157.5 ± 258.9	− 807.7 ± 122.1	− 174.0 ± 119.9
100 kVp	944.2 ± 229.2	− 498.7 ± 80.6	− 105.4 ± 100.8
120 kVp	713.1 ± 185.4	− 359.4 ± 68.7	− 94.7 ± 67.7
140 kVp	641.5 ± 141.6	− 286.4 ± 56.5	− 59.2 ± 52.0
Sn 100 kVp	326.4 ± 110.2	− 161.7 ± 74.5	− 58.2 ± 81.9
Sn 150 kVp	216.1 ± 76.2	− 85.9 ± 85.0	− 40.0 ± 89.4
**CBCT**			
60 kVp	941.0 ± 658.5	55.8 ± 271.2	− 73.1 ± 170.9
80 kVp	83.3 ± 93.6	73.8 ± 73.9	− 2.5 ± 72.5
102 kVp	73.2 ± 65.7	74.1 ± 59.9	− 3.2 ± 62.5
117 kVp	80.2 ± 53.2	73.8 ± 44.2	− 5.1 ± 51.6
133 keV	75.1 ± 52.8	75.2 ± 43.4	− 2.0 ± 43.5
**MDCT Sn 150 kVp**			
vs. MDCT 70 kVp	< .001	< .001	< .001
vs. MDCT 80 kVp	.003	< .001	< .001
vs. MDCT 100 kVp	< .001	< .001	< .001
vs. MDCT 120 kVp	< .001	< .001	< .001
vs. MDCT 140 kVp	< .001	< .001	.029
vs. MDCT Sn 100 kVp	.034	.023	.216
vs. CBCT 60 kVp	.017	.032	.940
vs. CBCT 80 kVp	.036	.008	.001
vs. CBCT 102 kVp	.039	.004	< .001
vs. CBCT 117 kVp	.027	.010	< .001
vs. CBCT 133 kVp	.033	.009	< .001
**CBCT 80 kVp**			
vs. MDCT 70 kVp	< .001	< .001	< .001
vs. MDCT 80 kVp	.001	< .001	< .001
vs. MDCT 100 kVp	< .001	< .001	< .001
vs. MDCT 120 kVp	< .001	< .001	< .001
vs. MDCT 140 kVp	< .001	< .001	< .001
vs. MDCT Sn 100 kVp	.007	< .001	< .001
vs. MDCT Sn 150 kVp	.036	.008	.001
vs. CBCT 60 kVp	.006	1	.156
vs. CBCT 102 kVp	.366	1	1
vs. CBCT 117 kVp	1	1	1
vs. CBCT 133 kVp	.925	1	1

CBCT, cone-beam computed tomography; kVp, kilovoltage peak; MDCT, multidetector computed tomography; Sn, 0.4 mm tin prefiltration.

### Analyzing the image quality

FOM analysis (Table 3) showed that UHR-CBCT protocols performed better than the dose-comparable UHR-MDCT protocols, owing primarily to relatively good ratings for protocols with low radiation exposure (except for a portion of 60 kVp scans, where image quality in bone tissue was considered insufficient for clinical use). The best protocol overall was the current clinical standard (80 kVp, 0.6 mean mAs per pulse). Despite favorable ratings, the higher-voltage protocols on both scanners achieved poorer FOM values due to their increase in radiation dose. Of note, tin-filtered UHR-MDCT scans received considerably better FOM values than standard UHR-MDCT examinations for bone image quality and artifact intensity. Homogeneous subset analysis of scan protocols based on tube voltage groups showed higher mean rank values for UHR-CBCT studies with at least 80 kVp regarding bone tissue delineation (Table 4) and artifacts (Table 5) (all *p* < 0.050). Interrater reliability of seven radiologists was substantial for bone and moderate for artifact assessment, as was indicated by Fleiss kappa of 0.652 (95% confidence interval 0.618–0.686; *p* < 0.001) and 0.570 (95% confidence interval 0.535–0.606; *p* < 0.001), respectively.

**Table 3:** Multi-observer assessment of bone image quality and artifact intensity.

Scan protocol	CTDI_vol_ [mGy]	Rating sum Bone	FOM Rank Bone	Rating sum Artifacts	FOM Rank Artifacts
**MDCT**					
70 kVp 50 mAs	1.3	7	#20	7	#14
70 kVp 97 mAs	2.7	11	#14	7	#22
80 kVp 122 mAs	5.1	14	#19	9	#23
70 kVp 199 mAs	5.3	12	#25	8	#24
Sn 100 kVp 898 mAs	7.5	21	#8	23	#5
Sn 150 kVp 146 mAs	8.2	24	#5	24	#7
100 kVp 113 mAs	9.8	20	#18	14	#21
80 kVp 241 mAs	10.2	21	#17	11	#25
120 kVp 101 mAs	14.1	28	#11	18	#18
140 kVp 96 mAs	18.9	26	#21	20	#20
100 kVp 227 mAs	19.5	26	#24	15	#27
80 kVp 489 mAs	20.3	18	#32	12	#29
120 kVp 202 mAs	28.1	35	#15	18	#26
140 kVp 192 mAs	37.7	31	#27	20	#28
100 kVp 453 mAs	39.3	28	#30	14	#32
120 kVp 405 mAs	54.0	37	#28	19	#30
140 kVp 383 mAs	75.2	35	#31	21	#31
**CBCT**					
60 kVp 0.6 mAs	2.3	7	#29	7	#19
60 kVp 1.2 mAs	4.5	14	#16	18	#4
80 kVp 0.6 mAs	4.5	21	#1	24	#1
102 kVp 0.6 mAs	9.1	24	#7	28	#2
60 kVp 2.5 mAs	9.5	16	#26	21	#12
80 kVp 1.2 mAs	9.9	28	#3	28	#3
117 kVp 0.6 mAs	13.6	33	#2	31	#6
102 kVp 1.2 mAs	16.3	33	#6	31	#9
80 kVp 2.5 mAs	17.4	32	#9	29	#11
133 kVp 0.6 mAs	21.6	35	#10	37	#8
117 kVp 1.2 mAs	23.6	41	#4	35	#10
102 kVp 2.5 mAs	34.6	35	#22	33	#16
133 kVp 1.2 mAs	36.3	42	#12	40	#13
117 kVp 2.5 mAs	44.1	46	#13	38	#15
133 kVp 2.5 mAs	61.0	46	#23	42	#17
Fleiss kappa (95% confidence interval; *p* < .001)		0.652 (0.618–0.686)		0.570 (0.535–0.606)	

CBCT, cone-beam computed tomography; FOM, figure of merit (sum of ratings^2^/CTDI_vol_); MDCT, multidetector computed tomography; Sn, 0.4 mm tin prefiltration.

**Table 4:** Homogeneous subset analysis for bone image quality based on tube voltage groups.

Bone image quality	Subset 1	Subset 2	Subset 3	Subset 4	Subset 5	Subset 6	Subset 7
MDCT 70 kVp	1.4						
CBCT 60 kVp	1.9						
MDCT 80 kVp		3.4					
MDCT Sn 100 kVp		4.5	4.5				
MDCT Sn 150 kVp			5.7	5.7			
MDCT 100 kVp			5.9	5.9			
CBCT 81 kVp				6.7	6.7		
CBCT 102 kVp					8.2	8.2	
MDCT 140 kVp					8.2	8.2	
MDCT 120 kVp						9.3	
CBCT 117 kVp							11.2
CBCT 133 kVp							11.5
Adjusted *p* value	.718	.162	.097	.515	.052	.349	.987

After differences between dependent nonparametric variables were ascertained with the Friedman test (*p* < .001), groups were compared pairwise with post-hoc tests and listed in order of ascending mean rank. The rank means that are listed under each subset are not significantly different from each other. In contrast, mean values that are not listed in the same subset differ significantly (adjusted *p* value for multiple comparisons < .050).

**Table 5:** Homogeneous subset analysis for image artifact intensity based on tube voltage groups.

Artifact intensity	Subset 1	Subset 2	Subset 3	Subset 4	Subset 5	Subset 6	Subset 7	Subset 8	Subset 9	Subset 10
MDCT 70 kVp	1.5									
MDCT 80 kVp	2.4	2.4								
MDCT 100 kVp		3.5	3.5							
CBCT 60 kVp		4.1	4.1	4.1						
MDCT 120 kVp			5.1	5.1	5.1					
MDCT 140 kVp				6.0	6.0	6.0				
MDCT Sn 100 kVp					6.9	6.9	6.9			
MDCT Sn 150 kVp						7.5	7.5			
CBCT 81 kVp							8.6	8.6		
CBCT 102 kVp								9.8	9.8	
CBCT 117 kVp									10.8	10.9
CBCT 133 kVp										11.7
Adjusted *p* value	.162	.066	.240	.287	.122	.360	.081	.094	.094	.052

After differences between dependent nonparametric variables were ascertained with the Friedman test (*p* < .001), groups were compared pairwise with post-hoc tests and listed in order of ascending mean rank. The rank means that are listed under each subset are not significantly different from each other. In contrast, mean values that are not listed in the same subset differ significantly (adjusted *p* value for multiple comparisons < .050).

## Discussion

In this experimental multi-observer study, we evaluated the potential of UHR-CBCT imaging in the setting of a present metal implant in comparison to a third-generation dual-source MDCT scanner with UHR option. To this effect, a total of 32 protocols with varying acquisition parameters were applied on a human cadaveric specimen. For tube potentials of 80 kVp and more, we were able to show that UHR-CBCT displayed superior performance regarding image artifacts as per objective analysis, and regarding image quality as per subjective analysis by seven radiologists. Furthermore, applied radiation dose was favorable in UHR-CBCT compared to UHR-MDCT protocols with similar image quality in bone and soft tissue.

The employed protocols for MDCT consisted of conventional CT imaging with tube voltage ranging from 70 to 140 kVp, as well as two protocols employing tin prefiltration for dedicated metal artefact reduction (Sn 100 kVp and Sn 150 kVp). In order to maintain comparability between CBCT and MDCT, all scans were performed in UHR mode and without employing dual-energy protocols. In other settings, spectral shaping via tin prefiltration has been shown to reduce applied dose considerably, especially in the case of obese patients when compared to dual-energy imaging and employment of virtual monochromatic reconstructions^21^. Notably and in contrast to UHR-CBCT, with current third-generation dual-source CT scanners, the necessity to employ an additional comb filter for UHR imaging effectively forces the choice between employing UHR acquisition and additional iterative MAR. While UHR-MDCT protocols employing spectral shaping via tin prefiltration significantly reduced metal artifacts compared to standard UHR-MDCT, UHR-CBCT with 80 kVp or more did allow for even superior artifact reduction and assessability of adjacent soft tissue. Also, regarding subjective image quality, UHR-CBCT protocols with tube voltage of 80 kVp or more displayed superior performance as compared to UHR-MDCT imaging. One explanation for the CBCT’s performance lies in its inherent superior dose efficiency which is realized by the system’s unique acquisition geometry: While regular gantry-based scanners operate with symmetric intervals between the X-ray tube and patient, as well as patient and detector, the employed CBCT’s two telescopic arms enable an asymmetric source-to-image distance with low magnification that improves spatial resolution by counteracting the limiting effect of the focal spot size^22^.

In light of recent publications demonstrating the potential and advantages of tableside UHR-CBCT imaging of the appendicular skeleton in trauma settings^19,22^, the reported findings are noteworthy. With regards to applied average dose, a recent meta-analysis by Nardi et al. states that CBCT promises a significant reduction over MDCT^23^. However, CBCT image quality is dependent on placement of the object of interest in the system’s iso-center due to the limited number of acquired image frames. This fact is more pronounced than in MDCT, potentially necessitating dedicated training and demanding explicit care by the radiographers.

CT imaging with metal implants present has been part of clinical routine for decades. However, artifact reduction in adjacent tissue is oftentimes of utmost importance, as even subtle findings such as fissure fractures require specific follow-up treatment up to secondary surgical revision. While high image quality is essential in these scans, dose reduction efforts can have a contrary effect on image quality. As conventional UHR-MDCT protocols were found to be clearly inferior and even UHR-MDCT protocols employing spectral shaping were not on par with UHR-CBCT imaging, the authors suggest future studies in clinical settings to further evaluate this promising imaging technique. In addition, dedicated studies quantifying the effect of the iterative metal artifact reduction algorithm with CBCT are mandated.

### Limitations

Some limitations ought to be mentioned regarding this study. For one, scans were limited to a single cadaveric specimen and to one type of implant. Thus, the impact of implant composition and size on resulting artifacts was not evaluated. Even though patients may well tolerate the required acquisition time of 14 s in CBCT due to comfortable tableside positioning, possible motion artifacts may be a limiting factor for clinical routine^23^. It lies in the nature of the study design, however, that motion artifacts did not play a role for this investigation. Furthermore, formalin fixation has been reported to invoke demineralization of the bone over time, impeding image quality irrespective of scanner and scan protocol^24,25^. Even though observers were blinded to the type of scan (UHR-CBCT vs. UHR-MDCT), a certain level of bias is conceivable due to the typical image impression of each imaging modality. Lastly, Multitom Rax (Siemens Healthineers) is not commercially available in all countries, therefore reproducibility of results may be limited.

## Conclusion

With the cone-beam CT scan mode of a gantry-free twin robotic X-ray system, ultra-high-resolution imaging of the appendicular skeleton in the presence of metal implants can be realized. Whilst the compared third-generation dual-source multidetector CT system does not support iterative metal artifact reduction algorithms in UHR-mode, achievable image quality and artifact reduction by means of cone-beam CT are superior to dose-comparable conventional UHR-multidetector CT and even multidetector CT protocols employing spectral shaping with tin prefiltration.

## Data Availability

The datasets used and/or analyzed during the current study are available from the corresponding author on reasonable request.

## Abbreviations

CBCT: Cone-beam computed tomography
CTDI_vol_: Volume computed tomography dose index for a 16 cm phantom (mGy)
MAR: Metal artifact reduction
MDCT: Multidetector computed tomography
ROI: Region of interest
UHR: Ultra-high-resolution
